# Therapeutic effects of different Atorvastatin doses on vulnerable plaques in coronary arteries assessed by intracoronary optical coherence tomography

**DOI:** 10.1097/MD.0000000000011718

**Published:** 2018-08-03

**Authors:** Honghua Ye, Shiqi Wang, Yewen Hu, Fuwei He, Jieqin Ju, Hanbin Cui, Xiaomin Chen

**Affiliations:** Department of Cardiology, Ningbo First Hospital. No 59 LiuTing street, Ningbo 315010, China.

**Keywords:** acute coronary syndrome fibrous cap, lipid core arc, optical coherence tomography, thin cap fibroatheroma

## Abstract

The aim of this study was to evaluate optical coherence tomography (OCT) as an assessment of the efficacy of atorvastatin treatment.

Twenty-four acute coronary syndrome (ACS) patients were allocated to conventional-dose (20 mg atorvastatin, n = 12) and intensive-dose (40–80 mg atorvastatin, n = 12) groups and correlations between changes in the OCT measurements and blood routine indexes were analyzed 9 months post-percutaneous coronary intervention (PCI).

Treatment with atorvastatin resulted in a significant increase in the target thin cap fibroatheroma (TCFA) fibrous cap thicknesses in both groups. The increase was bigger in the intensive-dose group than in the conventional-dose group (184.1 ± 57.4 μm vs. 125.1 ± 28.6, *P* = .005). The TCFA lipid core arc in both groups was significantly decreased compared with baseline (72.9 ± 29.3 vs. 127.6 ± 50.8, *P* < .01 and 74.6 ± 32.9 vs. 132.6 ± 51.3, *P* < .01, respectively). Correlation analyses showed an inverse relationship between low-density lipoprotein cholesterol (LDL-c) levels and the TCFA cap thickness, and a direct relationship between C-reactive protein (CRP) level and lipid core arc.

Statins significantly increased the TCFA fibrous cap thickness and reduced the lipid core arc, and OCT measurements accurately reflected the levels of blood LDL-c and CRP.

Trial registration: (Chinese Clinical Trial Registry) ChiCTR-IPR-17010874

## 1 Introduction

Acute coronary syndrome (ACS) is the main cause of adverse cardiac events in patients with cardiovascular disease (CVD). Pathologically, ACS is caused by atherosclerosis, which results from a build up plaques inside the arteries. The atherosclerosis plaques, which are very likely to rupture or erode and subsequently form a secondary thrombosis,^[1,2]^ are clinically termed as vulnerable plaques. Their main pathological characteristics include a thin fibrous cap, large lipid core, active inflammation, and positive remodeling. More than 75% of vulnerable plaques present as target thin cap fibroatheroma (TCFA), which is characterized by a fibrous thickness <65 μm and a lipid core area >40% in the entire plaque, with macrophage infiltration.^[3,4]^ Stabilization of vulnerable plaques is an effective method to prevent relapse of ACS and is an important area of current research.

Optical coherence tomography (OCT) is a medical imaging technique that uses near-infrared light to capture the structure of tissues at μm resolution. Its technical principle is similar to intravascular ultrasound (IVUS), with the difference being the employment of near-infrared light instead of sound waves. As an intravascular imaging technique, OCT currently provides the highest level of resolution at 10 μm in the axial direction and m□20 in lateral direction, which is about 10 times higher than IVUS. OCT produces accurate structural information of coronary arteries and has a degree of correlation with histological examination. It is widely used to assess a coronary atherosclerotic plaque *in vivo* and is the only intravascular imaging technique capable of accurately measuring the thickness of the TCFA fibrous cap.^[5,6]^ Some international preliminary studies have indicated that OCT can be used to observe the effect of statins on the stabilization of plaques in coronary arteries. However, these reports were based on retrospective observations,^[7]^ and there have been few prospective studies in which the effect of statins on the fibrous cap in lipid plaques was examined by controlling the statin dose. The aims of the present parallel group randomized controlled trial was to evaluate the effects of treatment with atorvastatin at conventional or intensive doses on vulnerable plaques in coronary arteries and to evaluate OCT utilization for assessment of the efficacy of statin treatment.

## 2 Patients and Methods

### 2.1 Patients

The study was approved by the ethics committee of the Ningbo First Hospital, and our study was performed in accordance with the Declaration of Helsinki regarding the ethical principles for medical research involving human subjects. Informed written consent was obtained from all patients. A total of 40 ACS patients diagnosed in the Cardiovascular Department of Ningbo First Hospital were recruited between 2011 and 2014. Among them, 16 were excluded because of either unfinished follow-ups or noncompliance of the treatment. Inclusion criteria were patients who were 15 to 80 years’ old and who had clinical diagnosis of ACS and lipid plaques besides culprit lesions. Exclusion criteria were patients who had taken statins before, had severe liver and kidney dysfunction, expected life span of less than a year, and thrombotic lesions. Patients whose target plaques required interventional therapy were also excluded.

A total of 24 patients participated in this prospective parallel grouped randomized controlled trial and were randomly allocated into an atorvastatin treatment conventional-dose group (n = 12) or an intensive-dose group (n = 12). The randomization process and allocation scheme were carried out 24 hours after OCT inspection. According to the list of random numbers generated by computer software SPSS17.0, patients were randomly divided into intensive statin group and normal statin group.

The diagnosis of patients was ST-segment elevation myocardial infarction (8 vs. 7), non–ST-segment elevation myocardial infarction (2 vs. 1), and unstable angina pectoris (UAP) (2 vs. 4 in the conventional and intensive dose groups, respectively).

### 2.2 Study design

All 24 patients received 300 mg each of aspirin and clopidogrel and were examined for non-culprit parts with OCT (C7-XR Dragonfly Intravascular Imaging Catheter, Lightlab Imaging Inc, Westford, MA) following post-percutaneous coronary intervention (PCI) treatment on culprit lesions. The location and number of TCFA were recorded and those patients with lipid plaques having the thinnest fibrous caps (thickness <65 μm) were selected as target plaques. The inclusion criterion of target plaques included: >2 quadrants of lipid composition; distinct appearance of fibrous cap; 30% to 70% stenosis detected by coronary angiography; ≥10 mm distance from the stent segment. The integrity of fibrous cap, the thickness of the thinnest fibrous cap (i.e., the shortest distance from the lumen side to the inner side of the lipid pool), and the lipid plaque arc were also recorded.

**Figure 1:**
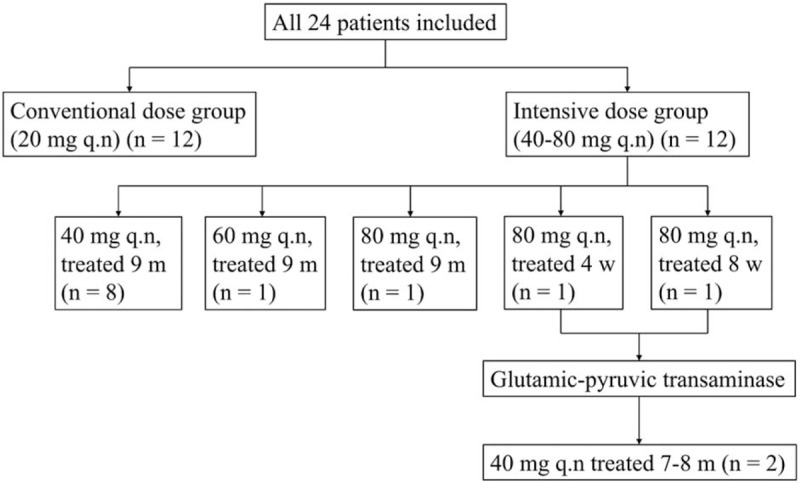
Diagram of treatment with different atorvastatin doses. Conventional-dose group: n = 12, 20 mg q.n. Intensive-dose group: n = 12, 40–80 mg q.n.

After PCI treatment, 12 subjects in the conventional-dose group received 20 mg atorvastatin q.n. for 9 months. In the intensive-dose group, 80 mg (maximum tolerable dose), 60 mg, and 40 mg atorvastatin q.n. were administered to 3, 1, and 8 subjects, respectively. However, in the case of 2 subjects who received 80 mg atorvastatin, due to the elevation of a liver enzyme, the dose was reduced to 40 mg at 4 and 8 weeks, respectively. These 2 subjects remained in the intensive-dose group. As a result, 12 patients were in this group and the average atorvastatin dose per subject was 45 mg q.n. (Fig. 1).

OCT was applied to review the target plaques in all 24 patients 9 months post-PCI and the changes in the fibrous cap thickness and lipid core arc were measured. The follow-up tracing of the target plaques was performed with OCT guided by coronary artery segment branch calcification and a stent placed in a coronary artery.

### 2.3 Laboratory measurements

Low-density lipoprotein cholesterol (LDL-C) (PVS colorimetry, Beijing Leagene Biotech.co., ltd, China) and C-reactive protein (Turbidimetry, Goldsite Diagnostics Inc., Shenzhen, China) were determined from sera of 5 mL fasting venous blood samples.

### 2.4 Statistical analyses and sample size and power calculation

SPSS Statistics for Windows (IBM Corp; Version 20.0, Armonk, NY) was used for data analysis. Normal distribution of quantitative data is shown as the mean ± standard deviation. A *t* test or a *χ*^2^ test was used for comparisons between the 2 groups and categorical data groups, respectively. Potential relationships among variants were analyzed by linear correlation analysis. A *P* value <.05 was considered to be statistically significant. According to the Easyfit study, intensive statin medication leads to fiber thickness increase of 73 μm, ordinary statin treatment group increased the 19μm, the standard deviation of 120 μm, α = 0.05, master degree is 0.8, using the pass 11.0 software to calculate sample size should be 11 cases in each group, assume that the losses in follow-up was 10%, the required sample size was 12 cases in each group

## 3 Results

The mean ages of participants in the conventional- and the intensive-dose group were 60.1 ± 11.1 and 60.1 ± 12.8 years, respectively, and the sex ratios were 9:3 and 9:3 (male: female), respectively. No significant difference (*P* > .05) was found between the treatment groups in clinical baselines of the subjects including age, sex, incidence of type 2 diabetes mellitus, hypertension, and hyperlipidemia (Table 1).

**Table 1:**
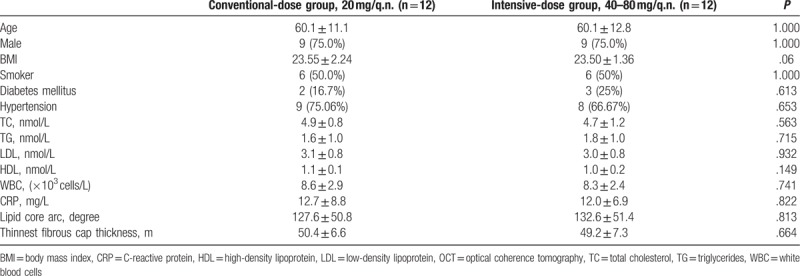
Comparison of clinical and OCT baseline characteristics between the conventional- and the intensive-dose groups.

No deaths, non-fatal myocardial infarction, target vessel revascularization, or UAP occurred in either group during the study period. The level of glutamic-pyruvic transaminase in 2 patients was increased from 8 U/L to 187 U/L and from 23 U/L to 263 U/L after 4 and 8 weeks of 80 mg atorvastatin treatment, respectively. However, 1 month after reducing the atorvastatin dose to 40 mg q.n. the levels of glutamic-pyruvic transaminase in these subjects returned to normal levels. No other adverse reactions, such as unendurable fatigue, muscle soreness or elevation of muscular enzymes were observed during the study.

**Figure 2:**
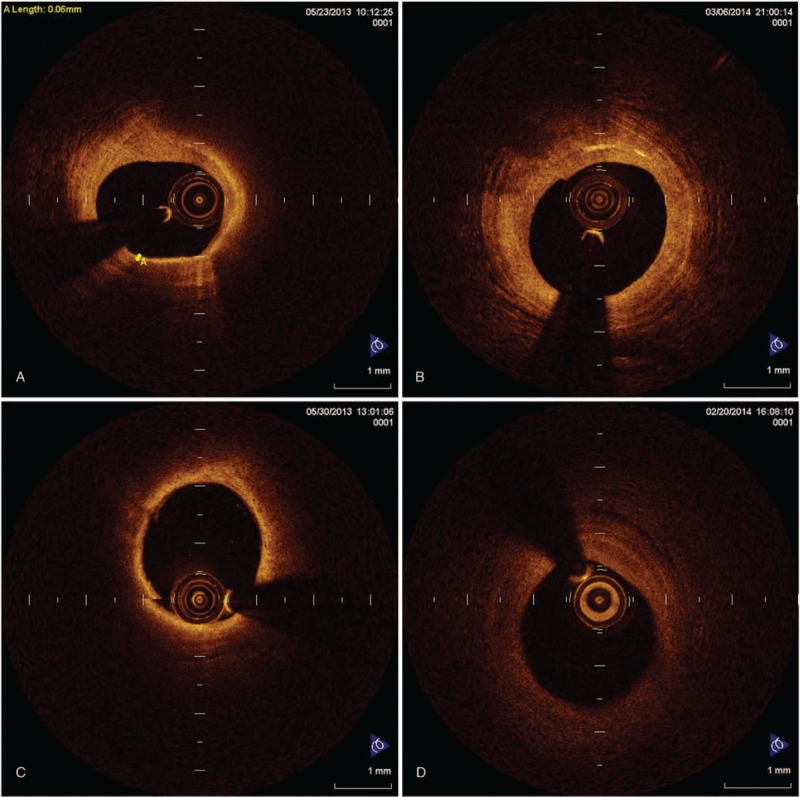
OCT images of TCFA plaques at baseline and after atorvastatin treatment. (A) A TCFA plaque at baseline with 60 μm of the fibrous cap thickness, 257 of lipid core arc and macrophage infiltration. (B) A TCFA plaque turning into a fibrous plaque at 9-month follow-up of 80 mg q.n. atorvastatin treatment with significantly increased fibrous cap thickness and reduced macrophage infiltration. (C) A TCFA plaque at baseline with 50 μm of the fibrous cap thickness, 190 of lipid core arc, and macrophage infiltration. (D) A TCFA plaque turning into a fibrous plaque at 9-month follow-up of 40 mg q.n. atorvastatin treatment with significantly increased fibrous cap thickness and reduced macrophage infiltration. OCT = optical coherence tomography, TCFA = target thin cap fibroatheroma.

OCT images showed that baseline lipid plaques were distinctively transformed to fibrous plaques after 9-month atorvastatin treatment (Fig. 2). The optical characteristics of lipid plaques were represented by heterogeneous properties and clouding margins with low signal reflection and high attenuation (low brightness) (Fig. 2A and C). In contrast, fibrous plaques appeared homogeneous with high signal reflection and low attenuation (high brightness) (Fig. 2B and D).

The fibrous cap thickness and lipid core arc of the target plaques were determined by OTC imaging analysis and compared between groups as well as baseline and 9-month follow-up measurements (Table 2). No significant difference in fibrous cap thickness of the target plaques was observed between the conventional- and the intensive-dose group at baseline. However, when compared to the baselines, at 9-month follow-up, the fibrous cap thickness was significantly increased both in the conventional- (from 50.4 ± 6.6 to 175.4 ± 36.0 μm, *P* < .001) and the intensive-dose group (from 49.2 ± 7.3 to 233.3 ± 88.4 μm, *P* < .001). Moreover, at 9-month follow-up the thickness of the TCFA fibrous cap in the intensive-dose group demonstrated a larger increase than that in the conventional dose group (184.1 ± 57.4 μm vs. 125.1 ± 28.6 μm, *P* = .005). In contrast, both the conventional- and the intensive-dose group showed a significant decrease in the lipid core arc of the target plaques at 9-month follow-ups compared with baseline (from 127.6 ± 50.8 to 72.9 ± 29.3 degree, *P* < .01 and from 132.6 ± 51.3 to 74.6 ± 32.9 degree, *P* < .01, respectively). However, no significant difference in the reduction of the lipid core arc was found between the conventional- and the intensive-dose group at 9-month follow-up of post-PCI (−54.7 ± 16.5 vs. −58.0 ± 27.4 degrees, *P* = .731).

**Table 2:**
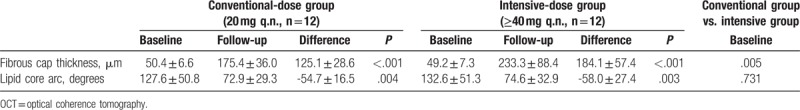
Changes in target plaque characteristics analyzed by OCT imaging in the conventional- and the intensive-dose group after 9-month atorvastatin treatment.

The relative change (absolute change/baseline value) in clinical blood routine indexes and in OCT measurements after atorvastatin treatment was calculated for both groups (Table 3). Compared with the conventional-dose group, the intensive-dose group exhibited a significantly greater increase in the thickness of the thinnest fibrous cap and a significantly greater decrease in LDL and total cholesterol levels. However, no significant difference between the groups was observed in the relative change in the degree of the lipid core arc and the blood CRP values. In addition, transformation from lipid plaques to fibrous plaques was observed at 9-month follow-up in 3 and 5 patients in the conventional- and the intensive-dose group, respectively. However, the difference in the frequency between the groups was not statistically significant (*P* = .386). Furthermore, the correlation analyses, as shown in Table 4, demonstrated that the relative change in the thickness of the thinnest fibrous cap negatively correlated with the relative change in the LDL level, whereas the relative change in the lipid core arc positively correlated with the relative change in the CRP level.

**Table 3:**
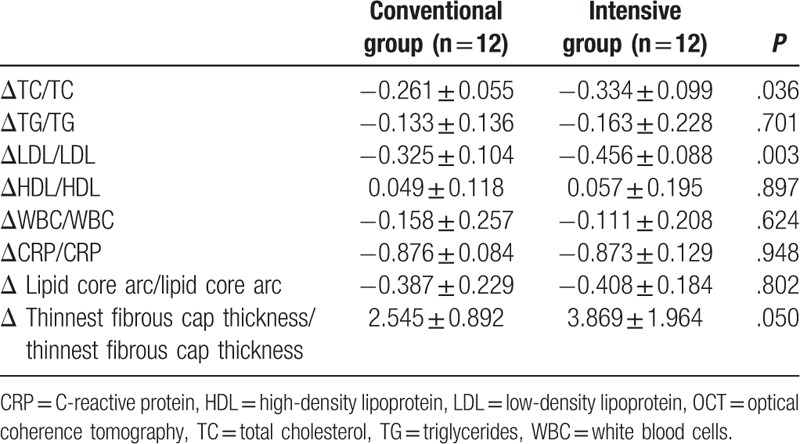
The relative change in blood indexes and OCT measurements after 9-month atorvastatin treatment.

**Table 4:**
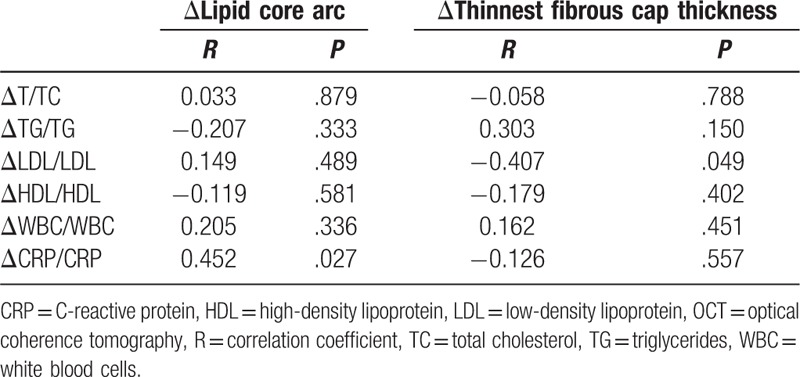
Correlation analysis between the relative change in OCT measurements of plaques and the relative change in clinical routine blood indexes in all 24 subjects.

## 4 Discussion

MIRACLE and PROVE-IT studies have shown that early treatment of ACS patients with atorvastatin at an intensive dose could significantly decrease the incidence of cardiac events.^[8,9]^ A ESTABLISH study reported a successful reversal of atherosclerotic coronary plaques by treatment with atorvastatin at a moderate dose (20 mg).^[10]^ Furthermore, a reversal study demonstrated that atorvastatin at an intensive dose (80 mg) rather than a conventional dose (40 mg) was more effective in the prevention of atherosclerotic plaque progression.^[11]^ However, the latter 2 studies assessed the plaque volume and composition by virtual histology intravascular ultrasound (VH-IVUS), which failed to measure accurately the fibrous cap thickness and lipid core arc of the plaques owing to the relatively low resolution of this method. In our study, the effects of atorvastatin at conventional and intensive doses were evaluated by directly measuring the TCFA fibrous cap thickness and lipid core arc utilizing OCT, and the therapeutic strategies with statins for ACS patients were further assessed.

Our study showed in ACS patients treated with atorvastatin at a conventional or intensive dose, that a significant increase in TCFA fibrous cap thickness and a decrease in lipid composition and macrophage infiltration was observed at 9-month follow-up. Especially the thickness of fibrous cap was found to be significantly increased in the intensive group compared with the conventional group. An Easyfit study reported that treatment with 20 mg atorvastatin compared to 5 mg significantly increased the fibrous cap thickness and decreased the lipid core arc and macrophage infiltration into atherosclerotic coronary plaques.^[12]^ We showed that treatment with 40 to 80 mg atorvastatin significantly increased the TCFA fibrous cap thickness compared with the 20-mg dose. However, no significant difference was found in the lipid core arc and the number of fibrous plaques transformed from lipid plaques, despite the fact that we used larger doses of atorvastatin. This finding requires verification by a further study in a larger cohort of patients. In addition, vascular inflammation plays an important role in the pathological changes in vulnerable plaques. A previous study has shown that the expression of inflammatory markers in ACS patients was reduced by statin treatment.^[13]^ The correlation between the thickness of the thinnest fibrous cap with the LDL level and the degree of the lipid core arc with the CRP level suggested that vulnerable plaques could be stabilized by the statins’ atherogenic lipoprotein-lowering, as well as anti-inflammatory, effects.^[14]^ During the course of our study, neither cardiac events nor adverse events such as muscular soreness, fatigue, and elevation of muscular enzymes were detected in both groups. A transient elevation of glutamic-pyruvic transaminase was observed in 2 patients in the intensive-dose group; however, the level returned to normal after the patients were treated with a reduced dose of atorvastatin. These observations indicated that the doses used in the present study were within the satisfactory safety dose-range of atorvastatin. A study with a larger cohort of patients and a longer study period is needed to evaluate further the clinical benefits of statins.

There were some limitations in this study because of a small number of recruited patients. For example, the atorvastatin dosage in the intensive group was 40 to 80 mg and averaged 48.3 mg; however, no subgroup analyses could be conducted because of insufficient patient numbers. Clearly, studies with a larger cohort of patients or a multicenter, randomized, double-blind, and placebo-control, should be conducted in the future. In addition, to address the clinical benefits of the increased thickness of the fibrous cap in vulnerable plaques, the 9-month follow-up period appeared to be insufficient. Therefore, a study with a prolonged duration needs to be carried out.

In summary, treatment with atorvastatin both at conventional and intensive doses significantly increased the thickness of the fibrous cap and reduced the lipid core arc in vulnerable plaques. Moreover, the increase in the thickness of the fibrous cap was significantly greater in the intensive-dose group compared to the conventional-dose group. The thickness of the thinnest fibrous cap inversely correlated with the LDL level, and the degree of the lipid core arc directly correlated with the CRP level, suggesting a dual effect of atorvastatin. OCT is likely to be an excellent intracoronary detection method and can be used to evaluate the therapeutic effects of statins.

## Author contributions

**Conceptualization:** Honghua Ye, Shiqi Wang.

**Data curation:** Honghua Ye, Shiqi Wang, Yewen Hu, Fuwei He, Jieqin Ju, Hanbin Cui, Xiaomin Chen.

**Formal analysis:** Honghua Ye, Shiqi Wang, Yewen Hu, Fuwei He.

**Funding acquisition:** Honghua Ye.

**Investigation:** Honghua Ye, Shiqi Wang.

**Methodology:** Honghua Ye, Shiqi Wang.

**Project administration:** Honghua Ye.

**Resources:** Honghua Ye, Shiqi Wang, Yewen Hu, Fuwei He, Jieqin Ju, Hanbin Cui, Xiaomin Chen.

**Software:** Honghua Ye, Shiqi Wang, Yewen Hu, Fuwei He, Jieqin Ju, Hanbin Cui, Xiaomin Chen.

**Supervision:** Honghua Ye.

**Validation:** Honghua Ye, Shiqi Wang, Yewen Hu.

**Visualization:** Honghua Ye.

**Writing – original draft:** Honghua Ye, Shiqi Wang.

**Writing – review & editing:** Honghua Ye.
